# IL-11 promotes Ang II-induced autophagy inhibition and mitochondrial dysfunction in atrial fibroblasts

**DOI:** 10.1515/biol-2025-1063

**Published:** 2025-03-11

**Authors:** Jun Wang, Qianyu Zhang, Yunjie Han, Jun Zhang, Nan Zheng

**Affiliations:** Department of Cardiovascular Medicine, Cangzhou Central Hospital, No. 16 Xinhua West Road, Cangzhou, Hebei, 061000, China

**Keywords:** Atrial fibrillation, atrial fibroblasts, Ang II, IL-11, oxidative stress, mitochondrial dysfunction, autophagy

## Abstract

This study aimed to investigate potential targets for the pathogenesis of atrial fibrillation to facilitate the development of effective treatments. Atrial fibroblasts were isolated and stimulated with 1 μM angiotensin-II (Ang-II) for 24 h. To increase interleukin 11 (IL-11) expression, overexpression plasmids were transfected into atrial fibroblasts. The role and the underlying mechanism of IL-11 in atrial fibrillation were examined by immunofluorescence, measurements of reactive oxygen species (ROS) and mitochondrial membrane potential (MMP), and western blotting assays. Results demonstrated that IL-11 was upregulated in Ang-II-elicited atrial fibroblasts. Ang-II treatment increases alpha-smooth muscle actin (α-SMA), ROS and MMP levels, and p62 expression but decreases microtubule-associated protein light chain 3 II/I (LC3 II/I) and Beclin-1 expressions in atrial fibroblasts. These effects were further amplified by IL-11 overexpression. Mechanistically, the mammalian target of rapamycin (mTOR) pathway expression was enhanced in Ang-II-induced atrial fibroblasts, which was further elevated by IL-11 upregulation. IL-11 facilitates Ang II-induced differentiation of atrial fibroblasts into myofibroblasts by promoting oxidative stress, mitochondrial dysfunction, and autophagy inhibition through the mTOR pathway.

## Graphical Abstract

**Figure:**
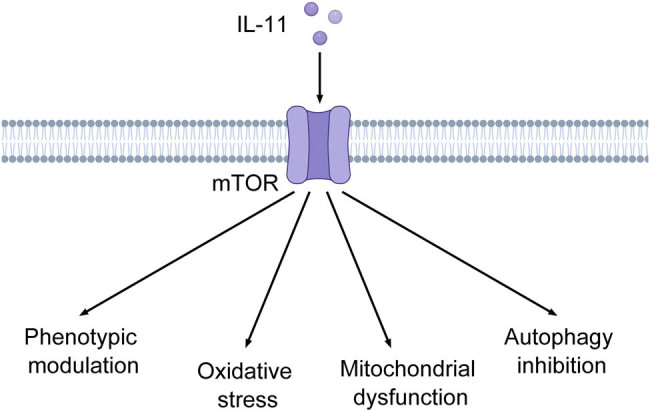

## 1 Introduction

Atrial fibrillation is one of the most prevalent arrhythmia diseases observed in clinical practice, significantly increasing the risk of morbidity and mortality. Its complications, including strokes and heart failures, markedly diminish patients’ quality of life [1]. Atrial fibrillation affects over 33 million people worldwide, and the incidence of the condition increases with age, with a prevalence of about 8.5% among patients over 65 [1,2]. Age is thus recognized as a major risk factor, alongside others such as genetic predisposition, hypertension, obesity, diabetes, male gender, endurance, exercise, and obstructive sleep apnea [2]. The primary pathological characteristic of atrial fibrillation is atrial structural remodeling, characterized by cavity enlargement and wall tissue fibrosis [1]. A key initial step in this process is the differentiation of atrial fibroblasts into myofibroblasts, often induced by endocrine peptide hormones such as angiotensin-II (Ang-II) and transforming growth factor β (TGF-β). Fibrotic lesions gradually develop in the local tissue by excessive myofibroblast proliferation, mobility, and the synthesis/deposition of extracellular matrix (ECM) proteins [3]. These fibrotic lesions impede the conduction of electrical pacing signals in the heart, creating a substrate for re-entry circuits and thereby elevating the risk of atrial fibrillation [3]. While the atrial fibrillation mechanism is not fully understood, pathogenesis, such as oxidative stress, mitochondrial dysfunction, and autophagy, has been implicated in its progression [4,5]. To develop effective treatment strategies, it is essential to identify potential targets involved in the disease’s pathogenesis.

Interleukin 11 (IL-11) belongs to the IL-6 family and has multiple functions. In response to inflammatory microenvironments, it upregulates or downregulates inflammatory responses, contributing to the development and progression of several diseases [6–8]. Recent studies have focused on the role of IL-11 in cardiovascular disease, particularly in cardiac fibrosis [8,9]. Persistent activation of the IL-11 signaling pathway has been linked to heart failure [10,12,13]. Furthermore, the administration of recombinant human IL-11 has been shown to increase the incidence of atrial arrhythmias in elderly patients [11] and aged rats but not in adult rats [12]. Elevated IL-11 levels have been observed in atrial fibrillation and are correlated with serum markers of fibrosis, making IL-11 a potential therapeutic target for atrial fibrosis [11]. However, in atrial fibrillation, IL-11 mechanisms remain unclear.

This study aimed to explore the mechanism of IL-11 in Ang-II-induced atrial fibroblasts. The results showed that IL-11 facilitated the phenotypic changes in myofibroblasts under Ang II stimulation. At the molecular level, IL-11 promoted Ang II-induced oxidative stress, mitochondrial dysfunction, and autophagy in atrial fibroblasts. Mechanistically, the expression of the mammalian target of rapamycin (mTOR) pathway was reduced in Ang-II-induced atrial fibroblasts, which was further declined by the upregulation of IL-11. Overall, the results indicated that IL-11 enhanced Ang II-induced atrial fibroblasts to differentiate into myofibroblasts by exacerbating oxidative stress, mitochondrial dysfunction, and autophagy via the mTOR pathway. This study provides valuable insights and identifies a potential therapeutic target for the diagnosis and treatment of atrial fibrillation.

## 2 Materials and methods

### 2.1 Atrial fibroblast isolation

Male wild-type C57BL/6J mice (6–8 weeks old) were obtained from Beijing Huafukang Biotechnology Co., Ltd (China). Mice were housed in a specific pathogen-free room for a week prior to the tests. Animal experiments were conducted in accordance with the Guide for the Care and Use of Laboratory Animals and authorized by the Animal Research Ethics Committee of Cangzhou Central Hospital. Atrial fibroblasts were isolated according to the previous study [12]. A 70% alcohol spray was used to disinfect mice’s bodies, and their hearts were collected by inhalation of excessive isoflurane (#R510-22, RWD, Guangdong, China). The left atrial was dissected into approximately 1 mm^3^ tissue blocks and immersed in digestive solution (DMEM [11965, Solarbio], including 0.25% trypsin [T1350, Solarbio], 1,500 U/mL collagenase type-II [40508ES76, YEASEN, Shanghai, China] and 15 mg/L BSA [36100ES25, YEASEN]) after rinsing off blood in pre-cooled phosphate buffer saline (PBS, P1020, Solarbio, Beijing, China) 3–4 times. Digestion was carried out for 20 min at 37°C in a 75 revolutions per minute (rpm) shaker. After gently dissociating cells with an elbow pipette, the supernatant was gathered and combined with 10% FBS (S9020, Solarbio) neutralizing DMEM. To harvest pellets, cell suspensions were percolated through a 70 μm mesh and centrifuged for 5 min at 1,000 rpm. Pellets were then re-suspended in DMEM with 10% FBS and hatched for 60 min at 37°C. Differential attachment was used to obtain atrial fibroblasts. The cellular morphology of isolated atrial fibroblasts was captured under an inverted microscope (Olympus, Tokyo, Japan). Atrial fibroblasts were sustained in DMEM with 10% FBS and 1% penicillin/streptomycin (P1400, Solarbio), and atrial fibroblasts were employed for second passage investigations.


**Ethical approval:** The research related to animal use has complied with all the relevant national regulations and institutional policies for the care and use of animals and has been approved by the Animal Research Ethics Committee of Cangzhou Central Hospital.

### 2.2 Cell treatment

Atrial fibroblasts were stimulated with 1 μM Ang-II for 24 h based on a previous report [12]. pcDNA vector plasmids carrying IL-11 sequences were also used to overexpress IL-11. Lipofectamine 3000 (L3000001, Invitrogen, Carlsbad, CA, USA) was then used to transfect them into atrial fibroblasts. Overexpression efficiency was evaluated using Western blots.

### 2.3 Immunofluorescence (IF) assay

IF experiments were conducted according to a previous study [13]. Atrial fibroblasts were seeded onto glass plates and cultured at 37°C with 5% CO_2_. After washing thrice with PBS, atrial fibroblasts were fixed with 4% paraformaldehyde (P1110, Solarbio) for 15 min at room temperature and rinsed with PBS thrice. Atrial fibroblasts were then incubated with BSA blocking buffer (SW3015, Solarbio), 0.2% Triton X-100 (T8200, Solarbio), and primary antibodies against vimentin (ab137321, Abcam, Cambridge, UK), von Willebrand factor (vWF, ab287962, Abcam), Desmin (ab15200, Abcam), and alpha-smooth muscle actin (α-SMA, ab7817, Abcam) overnight at 4°C. Subsequently, atrial fibroblasts were rinsed thrice with PBS and treated with donkey anti-Rabbit IgG H&L (Alexa Fluor^®^ 647) (1:1,000, ab150075, Abcam) or goat anti-mouse IgG H&L (Alexa Fluor^®^ 488) (1:1,000, ab150113, Abcam) for 1 h at room temperature. Atrial fibroblasts were stained with mounting medium, antifading (with DAPI) (S2110, Solarbio), and visualized using fluorescence microscopy (IX71, Olympus).

### 2.4 Reactive oxygen species (ROS) level examination

ROS levels were measured using dihydroethidium (DHE, S0063, Beyotime, Shanghai, China) based on the operating manual. Atrial fibroblasts were incubated with 5 μM DHE at 37°C and imaged under fluorescence microscopy.

### 2.5 Mitochondrial membrane potential (MMP) detection

MMP level was assessed using the Mitochondrial Membrane Potential Assay Kit with JC-1 (M8650, Solarbio) according to the operating manual. Atrial fibroblasts were mixed with 1 ml DMEM with JC-1 fluorescent dye and incubated for 20 min at 37°C. The cells were centrifuged for 5 min at 4°C at 600 × *g*. The supernatant was discarded, and atrial fibroblasts were resuspended in 1 × JC-1 buffer. Cell nuclei were stained with DAPI (C0065, Solarbio) and observed using a fluorescence microscope.

### 2.6 Western blotting

Atrial fibroblasts were lysed with RIPA lysis buffer (R0010, Solarbio) to isolate total proteins. Protein concentrations were measured with a BCA Protein Assay Kit (PC0020, Solarbio). Following electrophoresis using 10% sodium dodecyl sulfate-polyacrylamide gel electrophoresis (SDS-PAGE), protein samples (20 μg) were transferred to PVDF membranes (IPVH00010, EMD Millipore, Billerica, MA, USA). Membranes were treated with primary antibodies overnight at 4°C after blocking in 5% BSA blocking buffer (SW3015, Solarbio) for 1 h at room temperature. Membranes were then incubated for 1 h at room temperature with the secondary antibody goat anti-rabbit IgG H&L (HRP) (1:10,000, ab205718, Abcam). Bands were visualized using a BeyoECL Plus kit (P0018S, Beyotime), and the gray value was calculated using Image-ProPlus software (Media Cybernetics, Inc., Rockville, MD, USA). GAPDH acted as an internal reference. Primary antibodies included antibodies against IL-11 (1:5,000, PA5-95982, Invitrogen), peroxisome proliferator-activated receptor-gamma coactivator-1α (PGC-1α, 1:1,000, ab191838, Abcam), mitochondrial transcription factor A (TFAM, 1:1,000, ab47517, Abcam), LC3 I/II (1:1,000, 4108, Cell Signaling Technology, Inc., Danvers, MA, USA), Beclin-1 (1:1,000, ab62557, Abcam), p62 (1:1,000, ab91526, Abcam), phosphorylated mTOR (1:5,000, p-mTOR, ab109268, Abcam), mTOR (1:5,000, ab245370, Abcam), and GAPDH (1:2,500, ab9485, Abcam).

### 2.7 Statistical analysis

Data analysis was performed using SPSS 20.0 (IBM, Armonk, New York, USA). Data are presented as mean ± standard deviation (SD). The normality distribution of data was analyzed using the Anderson–Darling test, D’Agostino–Pearson omnibus normality test, Shapiro–Wilk normality test, and Kolmogorov–Smirnov normality test with Dallal–Wilkinson–Lilliefor *P* value, and the results showed that the distribution of all data was normal. The one-way analysis of variance (ANOVA) followed by a *Post Hoc* Bonferroni test was used to determine statistical differences. *P* < 0.05 indicates statistically significant differences.

## 3 Results

### 3.1 IL-11 promotes phenotypic changes in Ang-II-induced atrial fibroblasts

**Figure 1:**
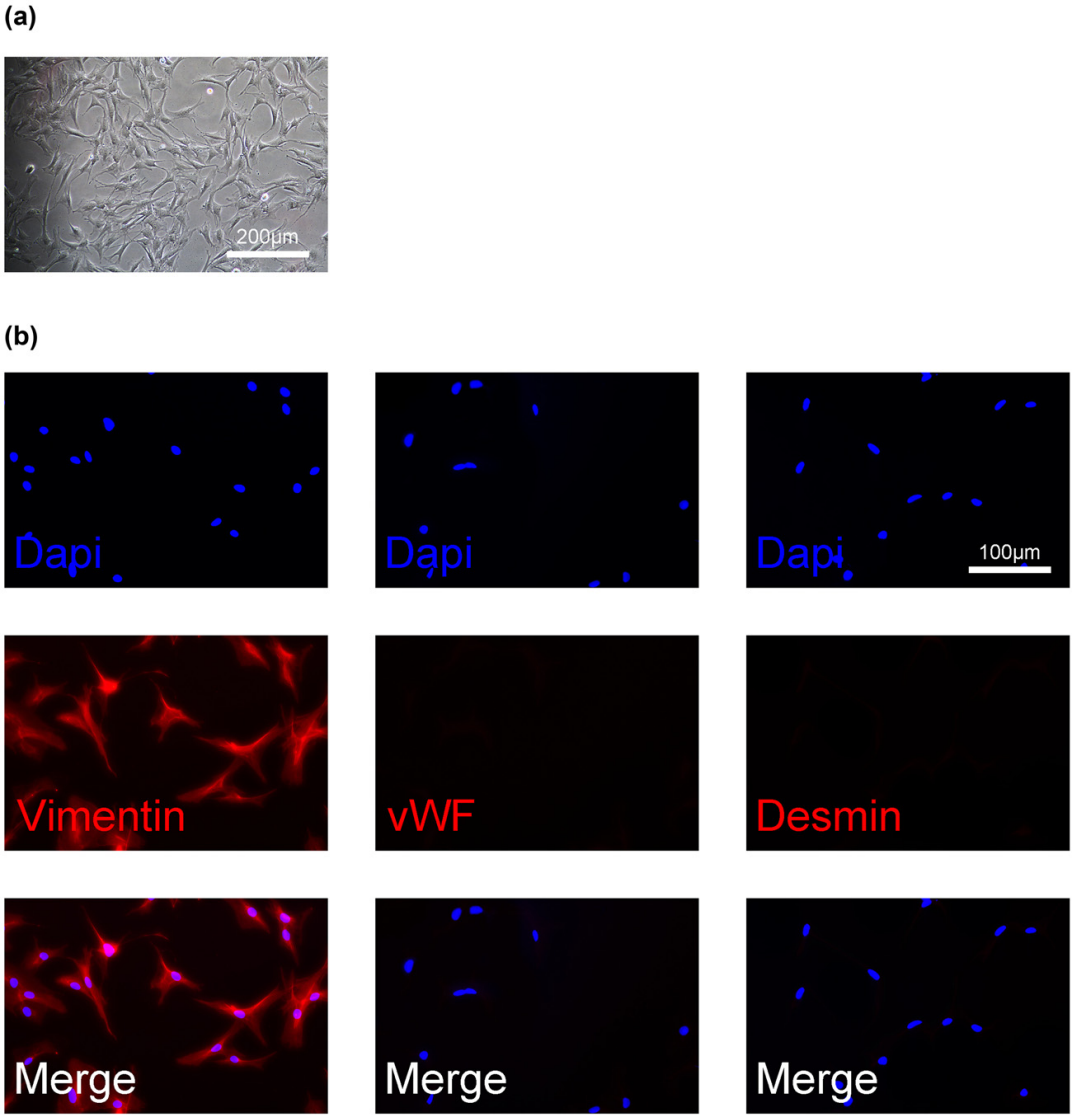
Identification of the isolated atrial fibroblasts. (a) The cellular morphology of isolated atrial fibroblasts was captured under an inverted microscope. (b) The expression of vimentin, vWF, and Desmin of isolated atrial fibroblasts was analyzed by IF.

**Figure 2:**
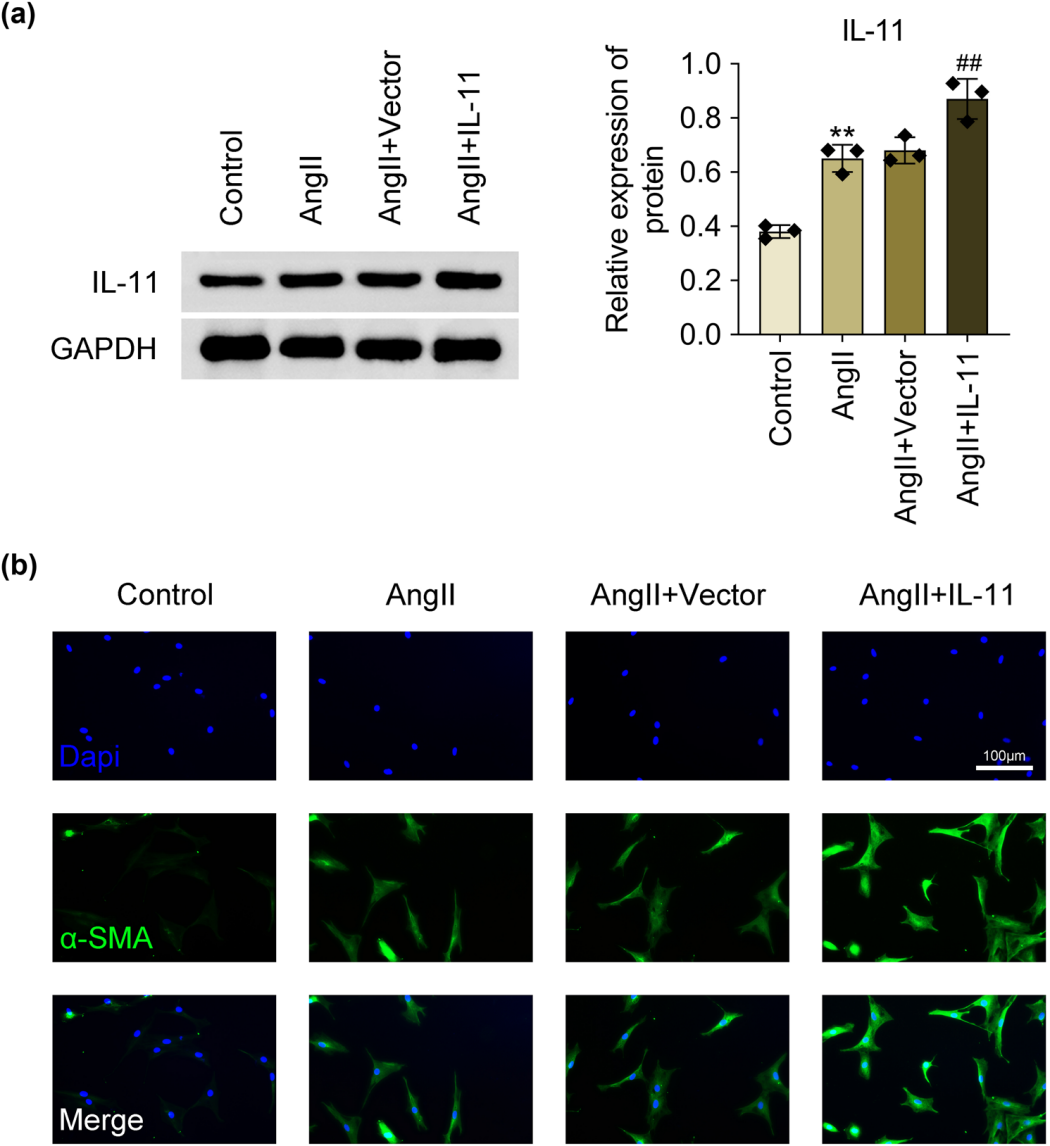
IL-11 enhances phenotypic changes in Ang-II-elicited atrial fibroblasts. Atrial fibroblasts were stimulated with 1 μM Ang-II for 24 h. IL-11 sequences were inserted into pcDNA vector plasmids and then transfected into atrial fibroblasts to overexpress IL-11. (A) IL-11 protein expression was detected by western blotting. Data were analyzed after normalization with GAPDH. **P < 0.01 vs. Control; ##*P* < 0.01 vs. Ang-II + Vector. *N* = 3. (B) α-SMA level was determined by immunofluorescence assays. Scale bar = 100 µm.

To investigate IL-11’s role in atrial fibrillation, atrial fibroblasts were first isolated. The cellular morphology of isolated atrial fibroblasts is shown in Figure 1a. Additionally, the expression of vimentin, vWF, and desmin of isolated atrial fibroblasts was analyzed. The results showed that vimentin was positively expressed in the isolated atrial fibroblasts, while vWF and desmin were negatively expressed in the isolated atrial fibroblasts (Figure 1b). The isolated atrial fibroblasts were then stimulated with Ang II for 24 h. IL-11 protein expression was significantly increased in Ang II-treated atrial fibroblasts compared to control cells (*P* < 0.01, Figure 2a). Overexpression of IL-11 in atrial fibroblasts was achieved via transfection with an IL-11 overexpression plasmid (*P* < 0.01, Figure 2a). Ang-II treatment resulted in a marked increase in α-SMA in atrial fibroblasts, which was further enhanced by IL-11 overexpression (Figure 2b). These findings indicate that IL-11 promotes phenotypic changes in Ang-II-induced atrial fibroblasts.

### 3.2 IL-11 enhances ROS production in Ang II-induced atrial fibroblasts

**Figure 3:**
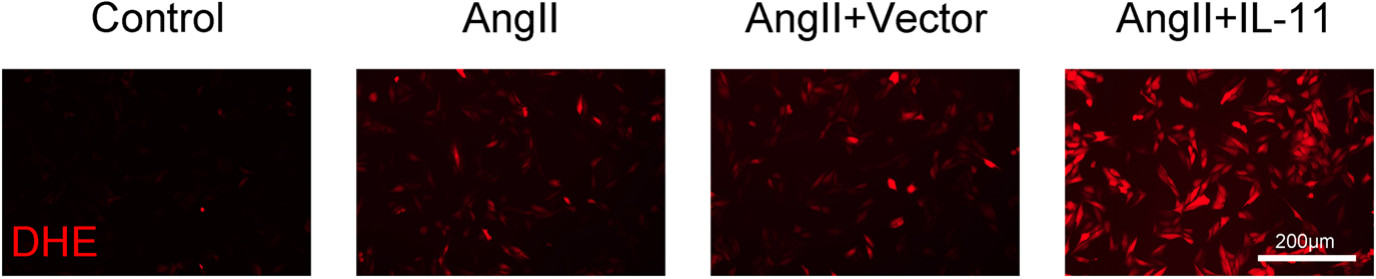
IL-11 increases ROS production in Ang II-induced atrial fibroblasts. ROS levels were examined after atrial fibroblasts were incubated with DHE for 30 min at 37°C. Scale bar = 200 µm.

IL-11’s role in atrial fibrillation pathogenesis, such as oxidative stress, mitochondrial dysfunction, and autophagy, was explored in Ang-II-induced atrial fibroblasts. To determine IL-11’s role in oxidative stress, ROS levels were measured in Ang-II-induced atrial fibroblasts. The results showed a significant increase in ROS levels in Ang-II-treated atrial fibroblasts, which was further distinctly enhanced by IL-11 overexpression (Figure 3). These findings suggest that IL-11 enhances ROS production in Ang II-induced atrial fibroblasts.

### 3.3 IL-11 intensifies Ang II-induced mitochondrial dysfunction in atrial fibroblasts

**Figure 4:**
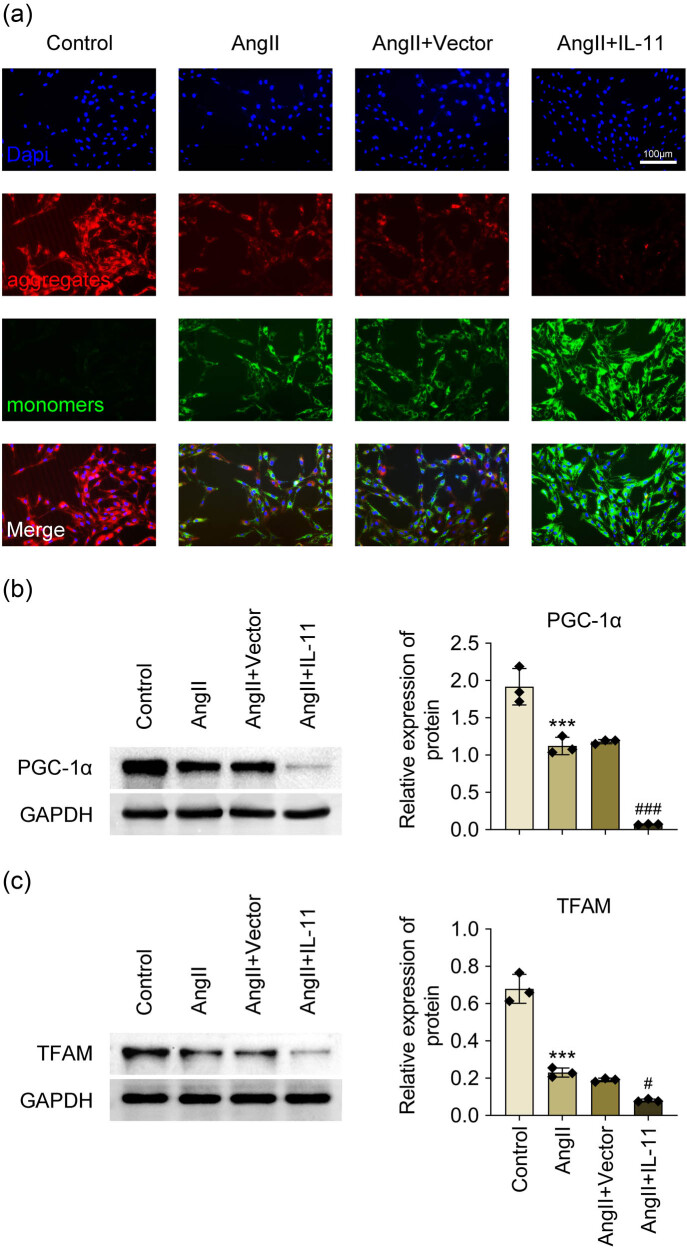
IL-11 reinforces mitochondrial dysfunction in Ang II-induced atrial fibroblasts. (a) MMP levels were examined after atrial fibroblasts were stained with JC-1. Scale bar = 100 µm. (b and c) Relative protein expression of PGC-1α and TFAM was detected by western blotting. Data were analyzed after normalization with GAPDH. ***P < 0.001 vs control; #*P* < 0.05 and ###*P* < 0.001 vs Ang-II + vector. *N* = 3.

IL-11’s role in mitochondrial dysfunction was probed in Ang-II-induced atrial fibroblasts. Ang II stimulation significantly increased the MMP level, which was further augmented by IL-11 overexpression (Figure 4a). Additionally, Ang-II treatment induced a significant decrease in the expression of PGC-1α and TFAM in atrial fibroblasts, which was further prominently reduced by IL-11 overexpression (Figure 4b and c). Altogether, mitochondrial dysfunction in Ang-II-stimulated atrial fibroblasts was aggravated by IL-11 overexpression.

### 3.4 IL-11 contributes to autophagy inhibition in Ang II-induced atrial fibroblasts

**Figure 5:**
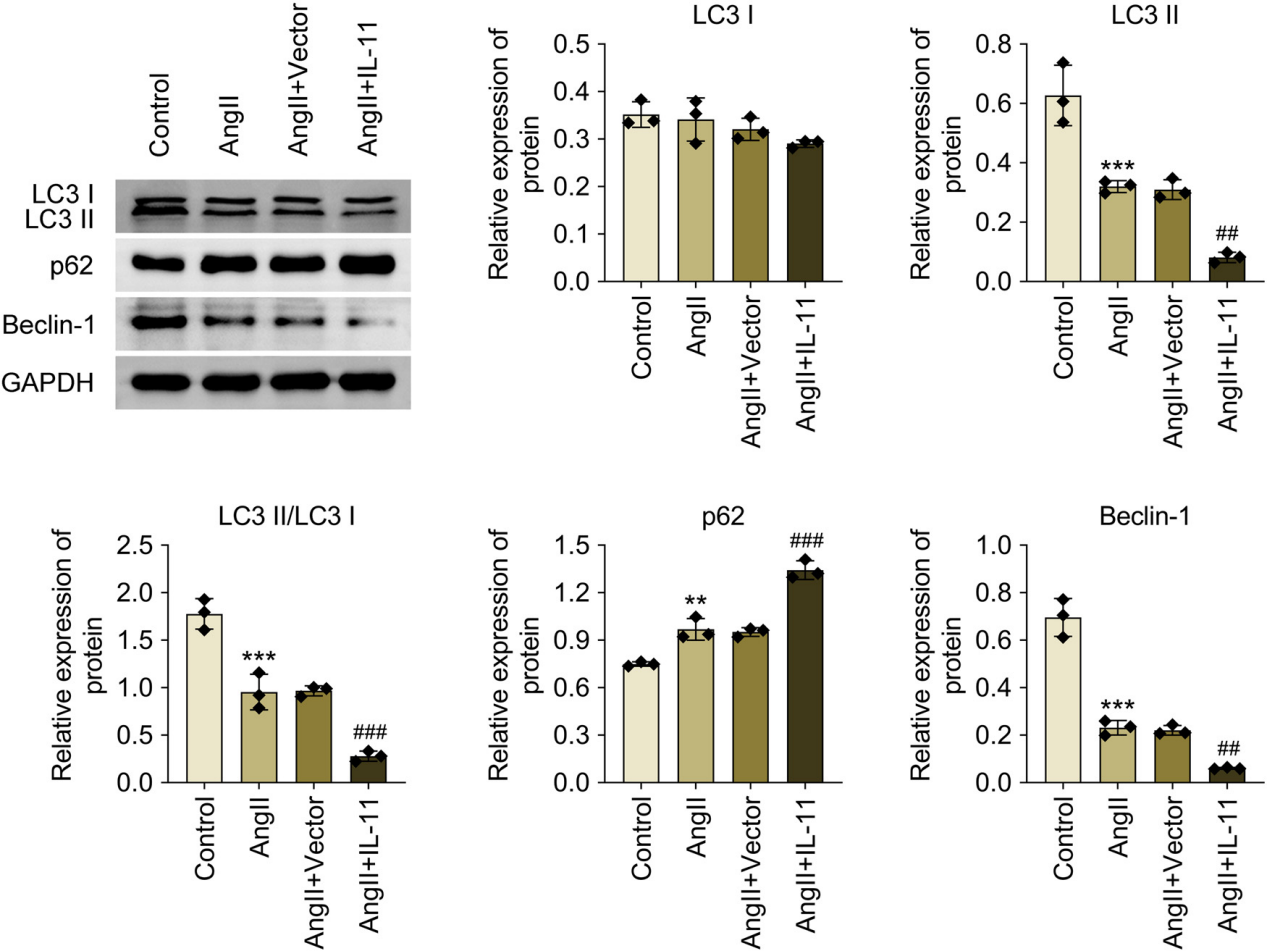
IL-11 facilitates autophagy inhibition in Ang II-elicited atrial fibroblasts. Relative protein expressions of LC3 I, LC3 II, LC3 II/I, p62, and Beclin-1 were determined by western blotting. Data were analyzed after normalization with GAPDH. **P < 0.01 and ***P < 0.001 vs control; ##*P* < 0.01 and ###*P* < 0.001 vs Ang-II + vector. *N* = 3.

The role of IL-11 in autophagy was investigated in Ang-II-induced atrial fibroblasts. The expression levels of LC3 II/I and Beclin-1 were significantly reduced in Ang-II-induced atrial fibroblasts, and these reductions were further diminished by IL-11 overexpression (*P* < 0.001, Figure 5). Conversely, IL-11 overexpression further enhances p62 expression in Ang II-induced atrial fibroblasts (*P* < 0.001, Figure 5). Hence, IL-11 promotes autophagy inhibition in Ang II-induced atrial fibroblasts.

### 3.5 IL-11 facilitates mTOR pathway activation in Ang-II-induced atrial fibroblasts

**Figure 6:**
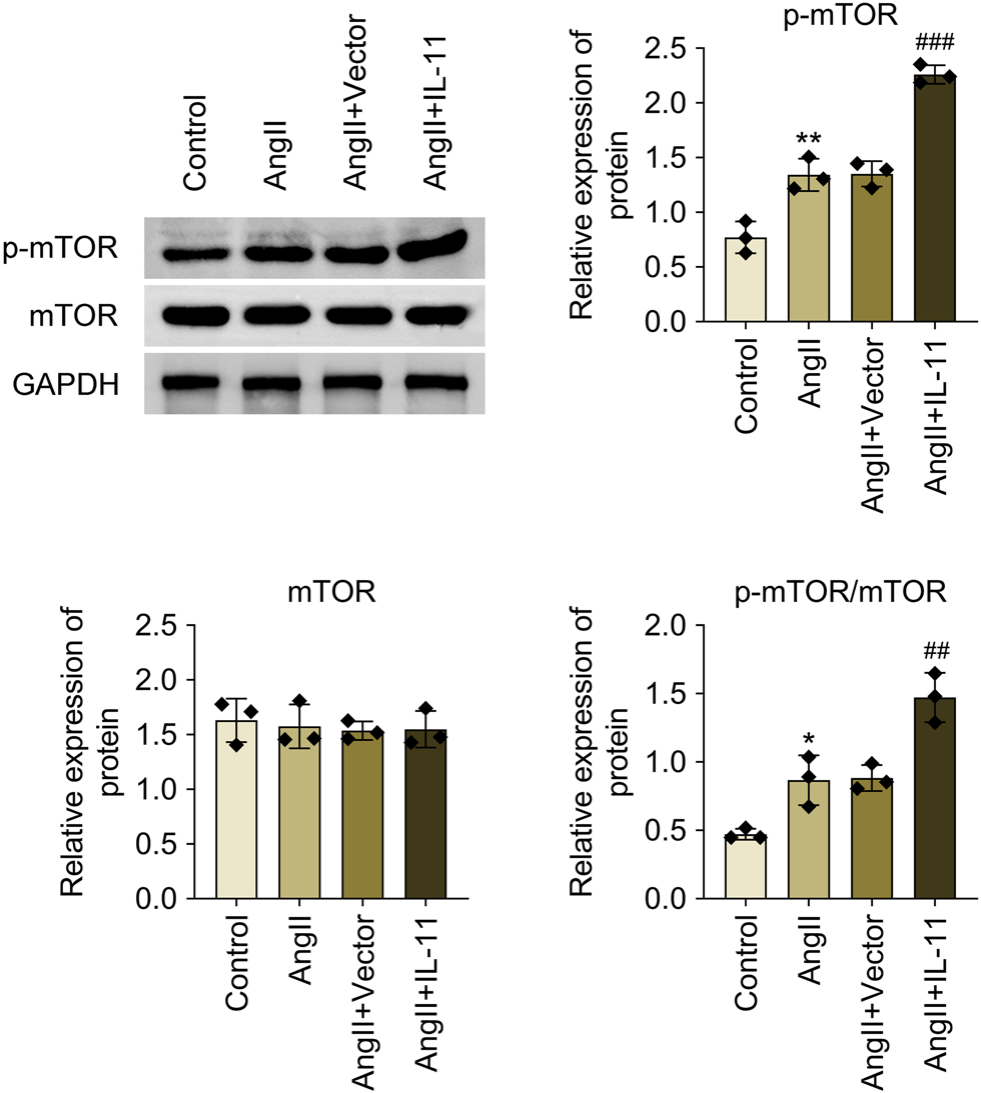
IL-11 enhances mTOR pathway activation in Ang-II-evoked atrial fibroblasts. Relative protein expressions of mTOR, p-mTOR, and p-mTOR/mTOR were quantified by western blotting. Data were analyzed after normalization with GAPDH. *P < 0.05 vs control; ###*P* < 0.01 and ###*P* < 0.001 vs Ang-II + vector. *N* = 3.

To investigate the potential mechanisms of IL-11 in atrial fibrillation, the activation of the mTOR pathway was examined in Ang II-induced atrial fibroblasts. The results demonstrated that p-mTOR/mTOR protein expression was enhanced in Ang-II-induced atrial fibroblasts, which was further elevated by IL-11 overexpression (*P* < 0.001, Figure 6). Collectively, IL-11 facilitates mTOR pathway activation in Ang-II-induced atrial fibroblasts.

## 4 Discussion

Atrial fibrillation is the most prevalent arrhythmia worldwide, contributing significantly to morbidity, mortality, and socioeconomic burden [4]. Current treatment approaches, such as catheter ablation techniques and antiarrhythmic medicines, have limited efficacy and side effects [14]. Developing gene-based therapies for atrial fibrillation presents an innovative and promising approach to treat atrial fibrillation [15]. In this study, we observed that IL-11 was upregulated in Ang-II-induced atrial fibroblasts, consistent with results from persistent atrial fibrillation patients and Ang II-treated fibrosis mouse models [11]. Ang-II treatment increases α-SMA, ROS, and MMP levels and p62 expression but decreases LC3 II/I and Beclin-1 expression in atrial fibroblasts. Atrial fibroblasts overexpressing IL-11 further confirmed the role of Ang-II in these indicators. Mechanistically, mTOR pathway expression was enhanced in Ang-II-induced atrial fibroblasts, which was further elevated by IL-11 upregulation. Overall, IL-11 promoted Ang II-induced atrial fibroblasts to differentiate into myofibroblasts by enhancing oxidative stress, mitochondrial dysfunction, and autophagy inhibition, involving in the mTOR pathway.

As a crucial mediator for atrial fibrosis, Ang-II abnormally stimulates atrial fibroblasts, driving their proliferation and transformation into a secretory phenotype known as myofibroblasts [3]. Myofibroblast phenotypes are characterized by α-SMA production. When myofibroblasts are produced, an overabundance of ECM is synthesized and deposited, eventually leading to fibrotic lesions in surrounding tissues [16]. These fibrotic lesions pose an increased risk of atrial fibrillation by obstructing the conduction of the heart’s electrical pacing signals [17]. Here, we found the increase of α-SMA in Ang-II-induced atrial fibroblasts was further enhanced by IL-11 overexpression, indicating that IL-11 promotes phenotypic changes in Ang-II-induced atrial fibroblasts. Similar results were also reported by Cong and colleagues, in which Ang II treatment fostered IL-11 expression and α-SMA intensity, while treatment with IL-11 antagonist reduced α-SMA-positive myofibroblasts in atrial tissue from the fibrosis mouse model [11]. Besides, IL-11 has been proven to transform fibroblasts into α-SMA-positive myofibroblasts in TGFβ1-stimulated human tracheal epithelial cells and primary rat tracheal fibroblasts [18]. Thus, IL-11 facilitates phenotypic changes in myofibroblasts in Ang-II-induced atrial fibroblasts.

Oxidative stress refers to an imbalance between ROS production and antioxidants [19]. ROS overproduction increases oxidative stress [20]. Increased ROS levels have been associated with atrial fibrillation [21]. Antioxidants, such as vitamin C, decrease the risk of postoperative atrial fibrillation [21,22]. This study discovered that increased ROS levels in Ang-II-stimulated atrial fibroblasts were further enhanced by IL-11 overexpression. However, IL-11 reduces ROS release in H_2_O_2_-induced PC12 cells [23]. Further research is needed to fully elucidate the role IL-11 plays in different diseases through its diverse molecular mechanisms. Notably, IL-11 promotes oxidative stress in Ang-II-induced atrial fibroblasts, but its broader impact on disease progression remains to be fully understood. Energy-producing organelles, mitochondria, play a crucial role in energy metabolism and the maintenance of myocardium redox balance [24,25]. Both experimental and clinical studies have demonstrated a reduction in ATP levels, loss of MMP, and fragmentation of the mitochondrial network, which causes contractile dysfunction and atrial fibrillation [26,27]. Therefore, several studies investigated mitochondrial dysfunction’s role in atrial fibrillation development [28,29]. Herein, Ang II stimulation resulted in an increase in MMP level, which was further enhanced by IL-11 overexpression. Assessing MMP is crucial in evaluating mitochondrial function. Besides, overexpression of IL-11 further aggravated the reduction in the expression of PGC-1α and TFAM in Ang-II-induced atrial fibroblasts. PGC-1α is a crucial transcriptional regulator of mitochondrial biogenesis, in which NRF1 acts as a principal target of PGC-1α. NRF1 activation raises TFAM expression, which primarily aids in mitochondrial DNA replication and maintenance [30]. Widjaja and colleagues [31] also reveal that IL-11 induces mitochondrial dysfunction in acetaminophen-evoked liver injury. Overall, IL-11 accelerates Ang II-induced mitochondrial dysfunction in atrial fibroblasts.

An autophagosome is formed when phagophores enclose proteins, RNA, and glycogen to initiate the breakdown process known as autophagy. Lysosome hydrolases then break down the inclusion. Autophagy is a physiological process by which cells maintain their homeostasis and regenerate their organelles. However, some cell stressors can also cause autophagy dysregulation. Cardiac autophagy is impaired in patients with postoperative atrial fibrillation after coronary artery bypass surgery [32]. Autophagy also promotes the profibrotic role of osteopontin in atrial fibrosis [33]. Additionally, a small-molecule-integrated stress response inhibitor is also reported to inhibit autophagy to assist its ameliorative role in atrial fibrillation [34]. Besides, autophagy-related genes have been identified as potential biomarkers and therapeutic targets in atrial fibrillation [35]. In this study, LC3 II/I and Beclin-1 downregulation and p62 upregulation were observed in Ang-II-induced atrial fibroblasts, which were intensified by IL-11 overexpression. Beclin-1 is crucial for autophagy’s initial step when it forms trimers with P13K and Atg14 and persistently attracts autophagy-related proteins [36]. By processing the LC3 precursor, Atg4 creates soluble LC3-I. LC3-II-PE is linked to phosphatidylethanolamine (PE) by Atg7 and Atg3, leading to lipid-soluble LC3-II-PE, which enhances autophagolysosome membranes until autophagolysosome formation [36]. Upon binding to the ubiquitinated protein, p62 joins forces with LC3-II, which is found in the cytoplasm, to form a complex that is ultimately broken down by lysosomes. Throughout the autophagic process, p62 will be continually eaten [36]. Sharma et al. [37] reported that mice treated with a long-acting IL-11 analog exhibit a decreased expression of Sqstm1, a marker of autophagy. Thus, IL-11 boosts autophagy inhibition in Ang II-elicited atrial fibroblasts, suggesting a possible treatment for atrial fibrosis by suppression of IL-mediated autophagy.

The conserved protein kinase, mTOR, connects the metabolic pathways in a wide range of species and is essential for cell metabolism, a fundamental biological process involved in cell growth, survival, and development [38]. The mTOR pathway also regulates other critical physiological functions such as autophagy, lipid synthesis, mitochondrial biogenesis, and cell survival [39]. mTOR deficiency and inhibition affect the heart differently, depending on the pathological and physiological state. In the embryonic and neonatal stages, the systemic or cardiac-specific mTOR knockout mice die in embryonic and neonatal stages mainly due to left ventricular dilatation and malfunction. This inhibits cell development, protein synthesis, mitochondrial activity, and sarcomere structure maintenance [39]. In unstressed settings, mTOR signaling is essential for the development of the cardiovascular system in the embryo and for maintaining cardiovascular integrity and function in the postnatal period [39]. Further, total genetic disruption of either mTORC1 or mTORC2 impairs the heart’s ability to adapt to mechanical and ischemia injury. This hinders compensatory cardiac hypertrophy in response to stress [40,41]. Inversely, partial and selective suppression of mTORC1 has been shown to provide cardioprotection to various cardiac pathological conditions, including pathological hypertrophy, cardiac aging, and genetic and metabolic cardiomyopathies [41]. This suggests the prospective therapeutic intervention of pharmacological suppression of mTORC1 for the management of cardiovascular ailments. This study found that p-mTOR/mTOR protein expression was enhanced in Ang-II-induced atrial fibroblasts, which was further elevated by IL-11overexpression. IL-11 is demonstrated to be secreted from damaged mammalian cells to signal through LKB1/mTOR in autocrine and paracrine [42]. Besides, Widjaja et al. [43] reported that activation of ERK/mTOR/P70S6K is essential for protein translation in TGFβ1-stimulated human cardiac fibroblasts, which is mediated by IL-11. The results from the same team show that IL-11 activates mTOR signaling and identify that the IL-11/mTOR axis acts as a signaling commonality in stromal, epithelial, and cancer cells [44]. Together, IL-11 facilitates mTOR pathway activation in Ang-II-induced atrial fibroblasts.

In summary, IL-11 was upregulated in Ang-II-induced atrial fibroblasts. IL-11 promotes the differentiation of Ang II-induced atrial fibroblasts into myofibroblasts by enhancing oxidative stress, mitochondrial dysfunction, and autophagy inhibition, processes that are involved in the mTOR pathway. However, several limitations should be addressed in future research. To further support the current study’s results, further studies should investigate the relevant indicators in more detail. This could include additional examinations of α-SMA expression using western blotting and immunohistochemistry, measurements of MDA, GSH, and SOD concentrations, and assessment of autophagy flux. Furthermore, the direct role of the mTOR pathway by using pharmacological blockers or gene interference can be explored in subsequent studies. In addition, the role of IL-11 in atrial fibrillation should be verified *in vivo*. Animal models, such as mice treated with Ang-II to induce atrial fibrillation, could be used to investigate the role of IL-11 in oxidative stress, mitochondrial dysfunction, and autophagy. Moreover, animal experiments could also confirm the involvement of the mTOR pathway using pharmacological blockers, thus solidifying the conclusions of this study. Overall, this study provides a better understanding and potential target for atrial fibrillation detection and treatment.
